# Septic Conditions of the Infantile Alimentary Canal, and Their Treatment

**Published:** 1898-03

**Authors:** 


**Septic Conditions of the Infantile Alimentary Canal, and their Treatment.** By F. W. Forbes Ross, M.D. Pp. viii., 138, xi. London: The Rebman Publishing Company (Ltd.). 1897.

To the subject of this volume the author has evidently given much careful thought, and as the book has some merit, certain parts will be found useful for reference. We are quite at one with the writer when he points out that success in the artificial feeding of infants and in the treatment of infantile gastro-intestinal disorders demands minute attention to details, trivial as some of these may appear. The chapter which he entitles "Sanitary Surroundings of Infantile Feeding in Health and Disease" is an eminently practical one, and the measures he there advises are founded on sound principles; and although the minutiæ may seem troublesome, they are certainly not unnecessary. We wish that the ideal plan that he gives of management of milch-cows, dairies, and milk distribution could be carried out: the necessity for some such system has recently been strongly borne in on the minds of residents in this neighbourhood. His directions for the treatment of intestinal diseases of children are good: the section on the employment of antiseptic remedies especially deserves praise; their value is abundantly confirmed by clinical experience. There is also a useful appendix giving modes of preparation of some simple foods, the dietary proper for infants at different ages, and directions for medicinal and dietetic treatment of intestinal disorders.

At the same time we must say that the clinical descriptions are too diffuse and not sufficiently clear, and that an embarrassing number of remedies are often advised. The author's style is throughout too vague and sometimes obscure, and there is a tendency to exaggeration and the redundant use of adjectives. We take exception to some of his statements, such as excoriation of the nates in infants "will kill, by the profound systemic derangement and exhaustion, if unattended," and to the arguments by which he infers that in some cases thrush is present somewhere in the alimentary canal, and acts as a cause of diarrhœa, even though the mouth and tongue show no signs of the fungus. The chapters on the gastric and intestinal secretions and on the bile do not add to the value of the book: some of the statements he makes on the properties of bile would certainly not be accepted by most physiologists. Rennin is misspelt "renin"; "enthusiastically hackneyed" we selected as a curious expression; and we are told that "castor oil . . . is perhaps after all the most valuable drug we possess in the treatment of sceptic [*sic*] conditions of the alimentary canal in infants." There is a section, which has little connection with the rest of the work, devoted to the advocacy of the use of orange juice in the treatment of typhoid fever; the reason for its use adduced is, that "organisms of a nature acting on sarcous matter will not to any great extent live in or act on a solution of vegetable acid." Amongst other favourable results of this treatment, the mental faculties were "capable of sustaining such light amusement as comic papers:" might not this be claimed by opponents of the treatment as evidence of mental deterioration? In spite of many faults, however, the book contains much good matter. It might be considerably shortened without detracting from its value.

